# Patient factors that affect trust in physicians: a cross-sectional study

**DOI:** 10.1186/s12875-018-0875-6

**Published:** 2018-11-29

**Authors:** Agnus M. Kim, Jaekyoung Bae, Sungchan Kang, Yeon-Yong Kim, Jin-Seok Lee

**Affiliations:** 1 Department of Health Policy and Management, Seoul National University College of Medicine, Seoul, Korea; 2 Graduate School of Public Health, Seoul National University, Seoul, Korea; 3 Big Data Steering Department, National Health Insurance Service, Wonju, Korea

**Keywords:** Trust, Patient-physician relationship, Republic of Korea

## Abstract

**Background:**

While trust in physicians has been rigorously investigated regarding its concept, measurement, and factors, the studies have mainly focused on the attributes of the physicians. This approach can lead to a limited understanding of trust in physicians as trust is based on the relationship, an interaction of both parties: patients and physicians. This study aimed to investigate the factors for trust in physicians among the Koreans by focusing on patients’ traits which are related to their subjective perceptions.

**Methods:**

A web-based survey was conducted between August and September 2016 among 1000 Korean adults aged 18 to 59 years. Survey participants were selected by a proportionate quota sampling based on age, sex and place of residence. The *t*-test and analysis of variance (ANOVA) were performed to examine the difference in trust in physicians among the different groups in each variable of patient characteristics. An ordinal logistic regression model was employed to examine the association between trust in physicians and patient attributes.

**Results:**

Negative health-related traits, such as stress and low self-rated health, were likely to lower trust in physicians, and women were less likely to trust physicians. The negative attitudes toward the current health care system were strongly associated with low trust in physicians. Meanwhile, recent experience of hospitalization or outpatient visit was positively associated with trust in physicians, and experience of not being able to use health facilities showed no significant association. These results suggest that trust in physicians is more likely to be lowered by negative perception than by the objective conditions or experience.

**Conclusion:**

In investigating the factors for trust in physicians, the patients’ predispositions, which make them less likely to trust physicians, should be considered. The attributes of the patients in Korea, which could negatively affect trust in physicians, need to be investigated in consideration of the recent changes in patient-physician relationships and the medical environment in Korea.

**Electronic supplementary material:**

The online version of this article (10.1186/s12875-018-0875-6) contains supplementary material, which is available to authorized users.

## Background

Trust is to believe that the other acts in the best interest of the one and to entrust oneself to the other. Given the significance that medical practice has on human life by concerning life and death directly [1] and the traits of medical practice, which often involve uncertainty [2] and irreversibility, trust is especially important in medical practice [3]. As medical practice involves patients’ cooperation, trust in physicians can contribute to the improvement of outcome of medical practice [4, 5]. Trust, on the other hand, can be an indicator of the patients’ satisfaction with medical practice and physicians [6]. As trust in physicians is deeply involved in medical practice and is also a faithful reflection of satisfaction about it, investigating trust in physicians would provide a clue about the patients’ attitudes toward the health care system, a large part of medical practice [7].

While trust in physicians has been rigorously investigated regarding its concept [3, 5, 8, 9], measurement [10], and factors, the studies have mainly focused on the attributes of the physicians, such as physician behaviors and performance [3, 5, 11–13]. Although patient characteristics have been included in some studies [4, 10, 13, 14], the traits were mostly limited to demographic characteristics, most of which were not strong predictors of trust or showed inconsistent results [6]. However, this approach can lead to a limited understanding of trust in physicians given that trust is based on the relationship, an interaction of both parties. Moreover, trust is a mental phenomenon based on perception [15] and is dependent on the subjects’ characteristics [16]. The mental determinants of trust may not necessarily result from the objective elements. For example, satisfaction, which is known to be deeply related to trust [17], is highly dependent on expectation rather than objective conditions of subjects [18]. In order to have an unbiased understanding of trust in physicians, the patients’ attributes, which can affect their subjective perception, need to be studied.

In that respect, Korea, where a large gap exists between objective health indices and the public’s perception of them, provides an intriguing condition for study of trust in physicians. Korea’s health care system has distinguishable features which seem mutually contradicting. While health status in Korea, as shown in high life expectancy at birth and low infant mortality, holds a high rank in the Organisation for Economic Co-operation and Development (OECD) member countries, the perceived health status of the Koreans places it low. And trust in physicians among the Koreans, in spite of the high level of health status they attained and high frequency of health care utilization, ranked low compared with the other countries [19, 20]. Investigating the factors for trust in physicians among Koreans will give clues in explaining these inconsistencies and will shed light onto how the patients’ characteristics relate to and influence the operation of the health care system in a country.

This study aimed to investigate the factors associated with trust in physicians among the Koreans. Rather than focusing on the physician behaviors, which were the main issue in the previous studies, we attempted to investigate patients’ traits which can affect their subjective perceptions, focusing on their health-related conditions, behaviors and attitudes as well as their demographic and socio-economic characteristics.

## Methods

### Participants

This study is based on the survey conducted with 1000 adults aged 18 to 59 years in Korea from August 28 through September 4, 2015 [21, 22]. Survey participants were selected among the panels who were certified by and registered in the survey company by providing their personal information and e-mail addresses. The selection was performed by a proportionate quota sampling based on age, sex and place of residence, and weights were applied in the analysis. The survey recorded information, including socio-demographic characteristics, health insurance, health status, health care utilization, health-related behaviors, attitudes toward the health care system, and trust in physicians. The survey was performed with web-based questionnaires developed by Research & Research, Inc., which had a certified quality control system [23]. This study was declared exempt from review by the institutional review board (IRB) of Seoul National University Hospital (IRB No. 1508–064-694). Online informed consents were obtained from all study participants before the survey.

### Dependent variable

The dependent variable in our study is general trust in physician. In most prior studies from the US, trust in physicians is based on the concept of my physician [1]. However, unlike in many western countries, the concept of my physician is rather unusual in Korea due to the practical absence of gate-keeping function [24]. In this study, we assessed general trust in physicians (subsequently referred to as trust in physicians). Subjects were asked, “How much do you trust physicians in Korea in general?” Response was categorized as a 5-point scale ranging from not at all (1) to very much (5). For the analysis, this 5-point scale was re-categorized into 3- point scale (not trust, ambiguous, trust).

### Independent variables

We selected variables which could affect trust in physicians on the basis of previous studies and our study hypothesis [4, 17]. These variables are classified into the following 5 categories: (1) socio- demographic characteristics such as sex, age (20–29, 30–39, 40–49, and over 50 years old), region (The Seoul Capital Area, Metropolitan areas, and Provinces), type of medical insurance (self-employed insured, employee insured, medical aid), education level (high school diploma or less, some college/ bachelor’s degree), monthly income (<$1900 USD, $1900 USD-$3800 USD, ≥ $3800 USD), and home ownership (privately owned, lease, monthly rent, provided for free), (2) health-related behaviors such as smoking, drinking, exercise, and stress, (3) health status such as perceived health status (very poor, poor, fair, good, very good), and stress, (4) health care utilization such as outpatient visits, hospitalization, and an experience of being unable to use hospitals/ clinics when necessary in the past 12 months, and (5) attitude toward the current health care system. The question for attitudes toward the current health care system was adopted from the Commonwealth Fund [25]. The questionnaire for the independent variables is provided in the Additional file 1: Table S1.

### Statistical analysis

We performed the *t*-test and analysis of variance (ANOVA) to examine the difference in trust in physicians among the different groups in each variable of patient characteristics. An ordinal logistic regression models was employed to assess the relationship between patient trust and patient characteristics. For data acquisition and analysis, IBM SPSS Statistics (version 22) and Stata (version 13) were used.

## Results

The results of univariate analysis between trust in physicians and the characteristics of the study subjects are presented in Table 1. Among the 1000 participants of the survey, 1000 participants (100%) completed the survey. The mean value of patient trust in physicians on a five-point scale was 3.30. Concerning demographic and socio-economic characteristics, the participants in lower income brackets scored lower trust than those in higher income brackets. There were no significant differences in trust in physicians among the groups with demographic or socio-economic characteristics like sex, age, education, area of residence, insurance type, and home ownership. In the case of health-related variables, stress and self-rated health status showed significant differences in trust in physicians; the participants with higher stress and the participants with lower self-rated health status showed lower scores. Health-related behaviors like smoking, drinking, or exercise did not show a significant difference among groups. Regarding health care experience, recent experience of outpatient visits or hospitalization and experience of being unable to visit hospitals or clinics when the visit was necessary, showed no significant difference. The satisfaction about the current health care system showed a marked difference among groups. The score was lower in groups with less satisfaction, and the group with the least satisfaction scored 2.56, which is the lowest among all the groups across the variables.

**Table 1:** General Characteristics of the participants

Characteristics	N	Mean	*p*-value
Total	1000	3.30	
Sex			
Male	520	3.32	0.12
Female	480	3.27	
Age group (years)			
20–29	227	3.31	0.21
30–39	241	3.21	
40–49	276	3.32	
≥ 50	256	3.34	
Region			
The Seoul Capital area	522	3.29	0.51
Metropolitan areas	199	3.34	
Provinces	279	3.27	
Medical insurance			
Self-employed insured	358	3.29	0.99
Employee insured	566	3.30	
Medical-aid beneficiary	76	3.30	
Home ownership			
Privately owned	630	3.32	0.25
Lease	204	3.26	
Monthly rent	140	3.20	
Provided for free	26	3.31	
Education level			
High school diploma or less	178	3.30	0.96
Some college/ bachelor’s degree	730	3.29	
Graduate/ professional degree	92	3.31	
Monthly household income			
<$1900	132	3.16	0.01
$1900 to ≤ $3800	385	3.27	
≥ $3800	483	3.35	
Smoking			
Never smoker	504	3.32	0.22
Daily smoker	217	3.22	
Intermittent smoker	90	3.26	
Former smoker	189	3.34	
Alcohol drinking frequency			
None	155	3.32	0.89
Once or less a week	606	3.29	
Two times or more a week	239	3.30	
Exercise frequency			
None	213	3.21	0.11
Once to twice a week	366	3.33	
Three to seven times a week	421	3.31	
Degree of stress			
Little or none	369	3.43	< 0.001
Moderate	515	3.27	
Extreme	116	3.00	
Self-rated health status			
Very good/ good	490	3.43	< 0.001
Normal	372	3.22	
Poor/ very poor	138	3.01	
Hospitalization in the past 12 months			
Yes	158	3.35	0.29
No	842	3.28	
Outpatient visit in the past 2 weeks			
Yes	363	3.31	0.49
No	637	3.28	
Being unable to use hospitals/ clinics when necessary in the past 12 months			
Yes	270	3.24	0.17
No	730	3.31	
Attitudes toward the current health system			
Positive	396	3.49	< 0.001
Negative	543	3.23	
Very negative	61	2.56	

The *t*-test and analysis of variance were used

We investigated the patient factors affecting trust in physicians (Table 2). Women were 0.67 times as likely to trust physicians as men. Daily smokers were 0.64 times as likely to trust physicians as never smokers. Stress was negatively related to trust in physicians, with the persons under severe stress being only half as likely to trust physicians as those under no or slight stress. Self-rated health was negatively related to trust in physicians; the persons thinking that they are in bad health were 0.29 times as likely to trust physicians as those in good health. Recent experience of outpatient visit was positively related to trust in physicians, and the experience of not being able to visit hospitals or clinics when necessary did not show any significant association with trust in physicians. Finally, the negative attitudes toward the current health care system were strongly related to lack of trust in physicians. The persons who had negative attitudes toward the current health care system were 0.43 (those with slightly negative attitudes) and 0.07 times (those with strongly negative attitudes) as likely to trust physicians as those who were satisfied.

**Table 2:** Ordinal regression analysis of trust in physicians and patient characteristics

Variables	Odds ratio	95% Confidence Interval	*p*-value
Sex (Ref: Male)			
Female	0.67*	0.49–0.91	0.01
Age group (Ref: 20–29, years)			
30–39	1.01	0.68–1.51	0.96
40–49	1.23	0.83–1.84	0.30
≥ 50	1.10	0.73–1.65	0.66
Region (Ref: The Seoul Capital Area)			
Metropolitan areas	1.05	0.75–1.36	0.79
Provinces	0.84	0.63–1.14	0.27
Medical insurance (Ref: Self-employed insured)			
Employee insured	0.97	0.73–1.28	0.82
Medical-aid beneficiary	1.11	0.66–1.86	0.69
Home ownership (Ref: Privately owned)			
Lease	0.87	0.63–1.21	0.41
Monthly rent	0.76	0.51–1.14	0.18
Provided for free	1.07	0.48–2.39	0.87
Education level (Ref: High school diploma or less)			
Some college/ bachelor’s degree	2.20	0.29–16.66	0.45
Graduate/ professional degree	1.21	0.84–1.75	0.30
Monthly household income (Ref:<$1900)			
$1900 to ≤ $3800	1.08	0.70–1.67	0.74
≥ $3800	1.25	0.79–1.95	0.34
Smoking (Ref: Never smoker)			
Daily smoker	0.64*	0.43–0.97	0.03
Intermittent smoker	0.90	0.54–1.49	0.68
Former smoker	1.00	0.68–1.46	0.98
Drinking alcohol frequency (Ref: None)			
Once or less a week	0.68	0.47–0.99	0.05
Two times or more a week	0.75	0.48–1.19	0.22
Exercise frequency (Ref: None)			
Once to twice a week	1.08	0.76–1.54	0.69
Three to seven times a week	1.05	0.74–1.49	0.79
Degree of stress (Ref: Little or none)			
Moderate	0.74*	0.56–0.98	0.04
Extreme	0.48**	0.30–0.75	< 0.01
Self-rated health status (Ref: Very good/ good)			
Fair	0.53**	0.39–0.70	< 0.01
Poor/ very poor	0.29**	0.19–0.45	< 0.01
Hospitalization in the past 12 months (Ref: None)	1.49*	1.03–2.16	0.03
Outpatient visit in the past 2 weeks (Ref: None)	1.37*	1.03–1.82	0.03
Being unable to use hospitals/clinics when necessary in the past 12 months (Ref: None)	1.00	0.74–1.35	0.99
Attitudes toward the current health system (Ref: Positive)			
Negative	0.43**	0.33–0.57	< 0.01
Very negative	0.07**	0.04–0.12	< 0.01

*p<,0.05. **p<,0.01, *R square* 0.104

## Discussion

This study investigated the relationship between trust in physicians and patient characteristics and explored the factors that affect trust in physicians. Health indicators related to bad health, such as frequent smoking, stress and low self-rated health, were found to lower trust in physicians. The recent experience of outpatient visits or hospitalization was associated with higher trust in physicians. The negative attitudes toward the current health care system were strongly associated with low trust in physicians. Our study results can be discussed in terms of two questions: 1) Which factors are associated with trust in physicians? 2) What are the factors that may lower trust in physicians among the Koreans?

First, women were less likely to trust physicians. This seems contrary to prior studies where female and male did not show a significant difference in trust in physicians [4, 13]. However, our results can be understood in line with trust in general. Studies of the generalized or interpersonal trust suggested that women were less trusting because they tend to fear risks and they, compared with males, are more likely to be discriminated against [26, 27]. In addition, several other attributes can explain the lower trust in physicians among women. First, women tended to have more negative perceptions of their own health and the current health care system [28, 29], which were shown to have a negative impact on trust in physicians in our analysis. Women’s higher preference for alternative medicine can also be a reason for lower trust in physicians. Compared with men, women in Korea are more dependent on oriental medicine [29, 30], a prevalent form of alternative medicine in Korea, which are practiced by different kinds of health personnel from physicians. Studies have shown that use of alternative medicine is negatively associated with trust in conventional medicine and its providers [31–33]. Considering the high proportion of oriental medicine, which accounts for more than 10% of the number of total outpatient visits in Korea [34], women’s higher dependence on oriental medicine could have negatively affected trust in physicians.

The factors, which could negatively affect health, were found to be strongly associated with low trust in physicians. These health-related factors can be divided into objective and subjective measures. Smoking is an established cause of bad health [35]. Stress, though also a well-known factor for ill-health [36], can be considered a subjective measure given that the survey question concerns the degree of stress the participant perceives. Self-rated health, though highly correlated with the objective health conditions, is also a subjective measure, which is not necessarily a direct reflection of the health status but rather a view or an estimation about one’s own health [37]. The negative relationship between negative health-related traits and trust in physicians can be due to a high likelihood that the persons in worse health conditions have negative experiences in health care and are less satisfied with it [38, 39].

However, considering that self-rated health and stress have subjective attributes which could result from one’s own perception, regardless of their objective conditions, the negative relationship between these variables and trust in physicians is suggestive of the association between lower trust in physicians and the propensity to develop negative attitudes independently of the objective conditions. This argument is convincing given that the level of perceived health among Koreans is the lowest among the OECD countries despite their high level of health conditions and high frequency of health services utilization [40]. These contradicting aspects indicate that the Koreans take a negative view of their own health conditions and are also quite likely to have negative attitudes toward the system or persons who provide the health services as much as they are dissatisfied. The fact that the negative attitudes toward the current health care system strongly lowered the likelihood to trust physicians also supports the possibility that the lower trust in physicians among the Koreans could be attributed to the patients’ negative attitudes, which are an inherent tendency rather than a consequence of poor external conditions.

As seen in the high correlation between trust in physicians and patient characteristics, which seem to be based on subjective perceptions, the low trust in physicians among the Koreans is likely to be attributed more to negative perception than external conditions. Patient experience is known to be the strongest predictor of the attitudes toward the health care system [38]. However, in our additional analysis, which is not presented in the table, the recent health care experience was not a significant factor in the attitudes toward the health care system. In addition, as presented in Table 2, the recent experience of outpatient visits or hospitalization was likely to increase trust in physicians. Furthermore, the experience of not being able to use health care facilities when necessary showed no significant relationship with trust in physicians. These findings strongly suggest that the low trust in physicians in Korea is less likely to result from their negative experiences concerning health care utilization. Our result is consistent with the result of a prior study that showed trust in physicians depended more on public opinion about the physicians and the generalized trust in a society than the individual experiences [2].

Based on the discussion hitherto performed, the factors that lower trust in physicians among the Koreans can be explained as follows: First, the low trust in physicians can be a result of the general negative attitudes, which are pervasive among other areas in Korean society [41]. For example, compared with other countries, the life satisfaction and self-rated safety scored lower among the Koreans [42]. As in the case of self-rated health, the low self-rated safety among the Koreans contrasts with the high score of public order and low crime rates in Korea. These negative attitudes can be indicative of the dissatisfaction resulting from higher standards or expectations or of the prevailing insecurities [43]. Second, the low trust in physicians among the Koreans can be a reflection of the low level of trust in Korean society. The generalized trust, which is toward other members of society, in Korea is low compared with other countries [42, 44, 45], and the institutional trust toward government or public institutions is also low [46, 47]. The relationship between a patient and physician is not only interpersonal but also institutional as physicians are a publicly authorized group whose right to practice is granted by government or institutionalized organizations. This characteristic brings trust in physicians under the influence of both types of general trust: interpersonal and institutional ones. The low trust in Korea can be attributed to the institutionalized corruption and the social inequalities [48], whose relationships to trust in physicians should be further investigated.

Lastly, the physician factors need to be scrutinized. If trust in physicians is more likely to result from public opinion than the individual experiences with the physicians [2], the low trust in physicians in Korea must be related to the negative public opinion about the physicians. Physician is still a highly sought-after job in Korea with decent earnings and respect from the society. However, the circumstances surrounding the physicians in Korea have been deteriorating for decades. This phenomenon is partly related to the world-wide trend in which the relationship between the physician and patients changes from the vertical to horizontal one. This change is, in part, due to the growing recognition of the rights of the patients [9] but is, on the other hand, an inevitable consequence of the overall increase in the level of education of the general population, which led to undermining the physicians’ authority that had stemmed from the exclusiveness of their professional knowledge.

However, these changes alone cannot explain the negative public opinion about the physicians in Korea. Physicians in Korea have been in conflict with the government in recent years concerning the payment methods and national health insurance fee schedule. The conflicts have been much publicized, and that caused the physicians to be recognized as a group which puts their interests first. In addition, the patient-physician relationship has become unfriendly. The amount of medical litigation has continued to grow in recent years [49], and patients’ violence toward physicians became so prevalent [50] that it eventually led to the recent passage of the bill that prevents violence toward physicians. These unfavorable conditions surrounding the physicians could have contributed to lowering trust in physicians and need to be further examined in future studies.

There are some caveats to be addressed in this study. First, unlike most prior studies of trust in physicians, the concept of trust in this study is for physicians in general and not for patients’ own physicians. In Korea, patients can freely visit physicians who practice in different types of health care facilities [24], and the concept of “my physician” is practically absent. Hence, in the Korean context, it is difficult to measure trust in “your” physician. Our survey accordingly stipulated “trust in physicians in general.” Second, although this study mainly concerns trust in physicians, the question concerning trust in physicians was a single question with the Likert scale. As a detailed description of trust was lacking, the interpretation of the results is likely to remain a general outline. However, what this study focused on was not a meticulous depiction of trust but the association between trust in physicians and patients’ characteristics. For that purpose, a simple question could serve as a comprehensive measure for assessing trust. Third, as this study is a cross-sectional study based on the short-term survey data, it has limitations in providing an explicit, causal inference which should be based on the temporal relationships among the variables. This should be recognized in interpreting our study results. Fourth, this study was on the basis of the survey results, and therefore the range of our statistical investigation was limited to the variables and their descriptions as presented in the survey. Given the limitations of the study data, we interpreted the study results in a way that connects and extends our results to those of the previous studies and the situation in Korea. As studies about the attitudes toward the health care system or the negative perceptions among the Korean population are lacking, qualitative studies need to be performed to support our study results. Despite these limitations, this study has its own value in that it investigated the patients’ factors for trust in physicians and elucidated that the factors were likely to be subjective ones based on perception rather than to be based on the objective measures or conditions. In addition, the social context presented in this study, concerning the general opinion toward the physicians in Korea, could have helped to understand the rather contradictory situations concerning the Korean health care system, which can be one explanation for the low trust in physicians in Korea.

## Conclusions

This study investigated the factors for trust in physicians among the Koreans in terms of the patients’ attributes. We found that the negative health-related traits, such as stress and low self-rated health, were likely to lower trust in physicians and that these traits were likely to be based more on the negative perception than on the objective conditions. The fact that the negative attitudes toward the current health system were the strongest factor that was likely to lower trust in physicians suggests that the subjective attributes of the patients could have contributed to lowering trust in physicians. The subjective attributes of the patients, which could negatively affect trust in physicians, should be investigated more in consideration with the recent changes in patient-physician relationships and the medical environment in Korea.

## Additional file

Additional file 1: The survey questionnaire for measuring trust in physicians and associated factors. (DOCX 17 kb)

## Abbreviation

OECD: Organisation for Economic Co-operation and Development
