# Supplementary material for: Patient factors that affect trust in physicians: a cross-sectional study
Source: BMC Fam Pract. 2018 Nov 29;19:187. doi: 10.1186/s12875-018-0875-6 (PMC6267873; doi:10.1186/s12875-018-0875-6)
Supplement: Supplementary file 1 — The survey questionnaire for measuring trust in physicians and associated factors. (DOCX 17 kb) [file 12875_2018_875_MOESM1_ESM.docx]

**Supplementary file 1. The survey questionnaire for measuring trust in physicians and associated factors**

1. Sex

1. Male 2. Female

2. How old are you?

1. 19-29 2. 30s 3. 40s 4. 50s 5. 60 and over

3. Where do you live?

1. Seoul 2. Pusan 3. Daegu 4. Incheon 5. Gwangju 6. Daejeon 7. Ulsan 8. Gyeonggi

9. Gangwon 10. Chungbuk 11. Chngnam 12. Jeonbuk 13. Jeonam 14. Gyeongbuk

15. Gyeongnam 16 Jeju

(For the analysis, 1,4, and 8 were calssified into Seoul Capital Area, 2 - 7 into Metropolitan area, and 9 – 11 into Provinces)

4. What kind of health insurance do you have?

1. Self-employed insured 2. Employee insured 3. Medical-aid beneficiary

5. What type of home ownership do you have?

1. Privately owned 2. Lease (Key Money Deposit, Jeonse) 3. Monthly rent 4. Provided for free

6. What is your highest level of education?

1. Middle school diploma or less 2. High school diploma 3. Some college/ bachelor’s degree 4. Graduate/ professional degree

1,2 into high school diploma or less

7. What is your family’s household monthly income?

1. < $950 2. $950 to $1,900 3. $1,900 to $2,850 4. $2,850 to $3,800 5. $3,800 to $4,750 6. $4,750 to $5,700 7. $5,700 and over

8. Do you smoke cigarettes now?

1. Every day 2. Intermittently 3. No, but smoked in the past 4. Never

9. How often do you drink alcohol?

1. Never in my lifetime 2. Never in the past year 3. Once a month 4. 2 to 4 times a month 5. 2 to 3 times a week 6. 4 or more times a week

10. How many days a week did you exercise for longer than 10 minutes?

1. 0 2. 1 3. 2 4. 3 5. 4 6. 5 7. 6 8. 7

11. How stressed do you feel in your daily life?

1. Extremely stressed 2. Moderately stressed 3. Slightly stressed 4. Not at all stressed

12. How is your health in general?

1. Very good 2. Good 3. Fair 4. Poor 5. Very poor

13. Have you ever been hospitalized in the past 12 months?

1. Yes 2. No

14. Have you ever used outpatient services in the past two weeks?

1. Yes 2. No

15. Have you ever been unable to use hospitals or clinics when necessary in the past 12 months?

1. Yes 2. No

16. How well do you think the Korean health care system is working in general?

1. On the whole, the system works pretty well and only minor changes are necessary to make it work better.

2. There are some good things in our health care system, but fundamental changes are needed to make it work better.

3. Our health care system has so much wrong with it that we need to completely rebuild it.

17. How much do you trust physicians in Korea in general?

1. Not at all 2. Not much 3. Neutral 4. Somewhat 5. Very much
